# Irinotecan-Induced Transient Dysarthria in a Patient With Metastatic Colorectal Cancer: A Case Report

**DOI:** 10.7759/cureus.54416

**Published:** 2024-02-18

**Authors:** Kim Alfred E Inting, Anthony N Piano

**Affiliations:** 1 Neurology, The Medical City, Pasig, PHL

**Keywords:** adverse event, cholinergic hyperactivity, colorectal cancer, dysarthria, irinotecan

## Abstract

We present a rare case of irinotecan-induced transient dysarthria in a 60-year-old female undergoing FOLFIRI (folinic acid (leucovorin), fluorouracil (5-FU), and irinotecan) chemotherapy for metastatic colorectal cancer. In eight out of the 12 cycles, an isolated, self-limiting "lingual dysarthria" with tongue stiffness consistently occurred during infusion and resolved promptly upon completion. Cranial imaging done during the initial episode and after the completion of the chemotherapy regimen were unremarkable. This temporal correlation suggests a drug-induced effect, proposed to be related to cholinergic hyperactivity associated with the drug's reversible inhibition of acetylcholinesterase. The hypoglossal nucleus, responsible for the motor function of the tongue, is selectively affected due to dense innervation by muscarinic cholinergic receptors (in comparison to other brainstem nuclei). This aligned with acute cholinergic syndrome common to irinotecan, and dysarthria might just be a rare manifestation of this common phenomenon. Its reversibility and atropine responsiveness support the cholinergic hypothesis. Clinicians should be mindful of this rare adverse event to avoid premature cessation of effective chemotherapy. Patient education on potential side effects and a comprehensive dysarthria work-up are crucial.

## Introduction

Irinotecan is a cytotoxic chemotherapeutic agent used to treat advanced-stage malignancies, particularly of gastrointestinal origin. It works in a cell cycle phase-specific (S-phase) mechanism and exerts its anti-tumor activity by inhibiting topoisomerase I, resulting in double-strand DNA breakage and cell death [1]. It reaches peak plasma concentration within 1-2 hours, and its metabolism is primarily hepatic (carboxylesterase enzymes) [2]. Common side effects include acute cholinergic syndrome (ACS) with nausea, vomiting, diarrhea, abdominal pain, and non-cholinergic symptoms such as myelosuppression, alopecia, and stomatitis. Cholinergic symptoms occur shortly after the beginning of infusion, are generally short-lived, and are rarely life-threatening [3]. On the other hand, central nervous system (CNS) adverse events related to irinotecan, particularly dysarthria, are rarely reported, and its incidence, mechanism, and pathophysiology still have not been fully elucidated. 

## Case presentation

We present the case of a 60-year-old female who presented with abdominal pain and a change in stool caliber and was later diagnosed with metastatic colorectal cancer. She promptly underwent resection (hemicolectomy) surgery and was initiated on systemic chemotherapy with FOLFOX (folinic acid (leucovorin), fluorouracil (5-FU), and oxaliplatin) regimen. Due to inadequate treatment response, this was shifted to FOLFIRI (folinic acid (leucovorin), fluorouracil (5-FU), and irinotecan) regimen every two weeks for 12 cycles over a period of six months and was given in the following manner: (1) concurrent administration of irinotecan 180 mg/m^2^ plus leucovorin 400 mg/m^2^ IV over two hours, (2) single IV bolus of 5-FU 400 mg/m^2^, and (3) a 48-hour continuous IV infusion at a dose of 5-FU 2400 mg/m^2^ via "easy pump."

During her second cycle of FOLFIRI, she had acute-onset dysarthria described as "stiffening of the tongue" with difficulty in enunciating words. This "lingual dysarthria" occurred in isolation with no other lateralizing neurologic deficits such as facial or pharyngeal muscle weakness attributable to dysarthria or other focal sensory or motor abnormalities. Upon careful review, this occurred 10-15 minutes after initiation of irinotecan infusion. There were no signs of hypersensitivity reaction such as rashes, chest tightness, or difficulty breathing as well as absence of the more common cholinergic adverse events such as sweating, flushing, hypersalivation, or vomiting. The dysarthria resolved spontaneously after the completion of irinotecan infusion, lasting approximately 120 minutes during this initial episode. Subsequent diagnostic work-up with contrast-enhanced cranial magnetic resonance imaging (MRI) was negative for acute infarct, hemorrhage, metastatic lesions, or leptomeningeal enhancement. Due to her underlying malignancy and increased risk for thrombotic-ischemic events, the patient was treated as a case of transient ischemic attack (TIA) and started on aspirin for secondary stroke prevention. Close observation for any recurrence of the symptom was then done.

Repeated episodes of lingual dysarthria of the same characteristic were observed on subsequent cycles, with documented temporal association of the drug's administration and development of dysarthria (time-locked events) becoming more evident (see Table 1). These events consistently occurred during the drug's infusion and promptly resolved after its completion. However, this did not occur on some of the cycles (first, fifth, ninth, 11th), and the duration of dysarthria progressively became shorter as the cycles progressed. As early as the third cycle, the case was identified as irinotecan-induced dysarthria, and aspirin (for stroke prevention) was already discontinued since it was no longer indicated. Upon joint discussion with the patient and family, chemotherapy was continued and completed as the side effect of concern was tolerable and reversible; moreover, the potential benefit outweighed the risk. After completion of the chemotherapy regimen (FOLFIRI) and on subsequent follow-ups, there were no recurrences of the dysarthria. Repeat cranial MRI with contrast imaging after completion of the chemotherapy regimen still revealed negative findings. Thus, this drug-induced dysarthria was deemed to be self-limited with no long-term consequences.

**Table 1:** Time-locked events of dysarthria and irinotecan infusion

	Onset (after irinotecan initiation)	Resolution (after irinotecan completion)	Duration of dysarthria
3rd cycle	10 minutes	30 minutes	2 hours and 20 minutes
4th cycle	10 minutes	25 minutes	2 hours and 15 minutes
6th cycle	30 minutes	30 minutes	2 hours
7th cycle	20 minutes	15 minutes	1 hour and 55 minutes
8th cycle	30 minutes	10 minutes	1 hour and 40 minutes
10th cycle	30 minutes	5 minutes	1 hour and 35 minutes
12th cycle	35 minutes	5 minutes	1 hour and 30 minutes

## Discussion

Dysarthria is defined as an impairment of speech caused by an alteration of the strength and control of muscles of articulation (facial, lingual, pharyngeal) causing slurred speech that is difficult to understand. Common causes include stroke, trauma, and brain tumors. Less common causes are medication-related, including chemotherapeutic agents but rarely with irinotecan [4].

Adverse reactions associated with irinotecan are most commonly related to cholinergic toxicity, mediated through irinotecan's rapidly reversible inhibition of acetylcholinesterase (AChE) and increase in acetylcholine activity [5]. This explains the transient nature of these adverse reactions and its prevention with co-administration of atropine, an anticholinergic [5]. Although transient dysarthria is not reported as a side effect of irinotecan's prescribing information sheet, this event may still be related to the same "cholinergic hyperactivity" as the main culprit. Some authors propose the selective overstimulation of the hypoglossal (XII) nucleus in the medulla by acetylcholine since it is densely innervated with muscarinic cholinergic receptors relative to the other brainstem nuclei [6-9]. This hypothesis may explain the production of "tongue stiffness" observed in our case causing lingual dysarthria. Likewise, this also explains the transient nature of the symptom and its prompt resolution after discontinuation of the culprit drug, reminiscent of the other common cholinergic adverse reactions (sweating, flushing, hypersalivation, abdominal pain, vomiting, and diarrhea). Simply put, transient dysarthria associated with irinotecan may be a rare manifestation of the more common ACS.

Risk factors associated with the development of irinotecan-induced dysarthria are still unknown but may be related to variations in muscarinic cholinergic receptor densities in the hypoglossal nucleus between individuals. Most of the brainstem nuclei containing these receptors were enriched in high-affinity agonist-binding sites, although it was consistently the most abundant and with the highest concentrations in the hypoglossal nucleus, as shown in microscopic studies [6-7].

On review of reported cases [8-10], dysarthria was similarly reported to be reversible and had quick resolution (ranging from two to seven hours). It mostly occurred during the first cycle usually within 90 minutes of drug infusion, although it may occur in subsequent cycles. Imaging of the brain, such as MRI or CT scan, failed to show any evidence of acute CNS abnormalities. Some cases reported improvement and resolution with atropine administration, further suggesting the relationship between irinotecan-induced transient dysarthria and cholinergic hyperactivity. However, not all cases respond with atropine, raising questions about its utility in treatment. Resolution was also independent of other strategies adopted, such as discontinuing infusion, reducing the infusion rate or dosage, or administering some intravenous fluids. Similarly, its effect is not cumulative, and severity did not increase upon re-administration of irinotecan in subsequent cycles. Taken together, these factors probably suggest an idiosyncratic adverse reaction. Undoubtedly, additional studies are warranted to elucidate the pharmacodynamic mechanisms behind this rare adverse event. However, a unique aspect observed in this case, not observed in the previously published cases, is the gradual reduction in the duration of dysarthria episodes as the regimen progressed, a phenomenon that remains uncertain to the authors.

## Conclusions

Clinicians must be cognizant of this rare adverse event to prevent unnecessary discontinuation of a potentially effective drug. Irinotecan-induced lingual dysarthria seems to be transient, self-limiting, and with no long-term neurologic sequelae. Current data and observations lead us to believe that it is related to the cholinergic hyperactivity of the hypoglossal nucleus due to the drug's reversible inhibition of AChE. Proper patient education prior to initiation of the agent is important to set expectations and avoid unnecessary stress for the patient and family. Of course, this is a diagnosis of exclusion, and the initial work-up should include ruling out other causes of dysarthria such as stroke or brain metastases, especially in the background of a known malignancy.
